# Synchronous Acute Acalculous Cholecystitis and Appendicitis Due to *Salmonella* Group D: A Rare Case Report From China and Review of the Literature

**DOI:** 10.3389/fmed.2020.00406

**Published:** 2020-08-18

**Authors:** Yan Zhao, Lijun Zhang, Fanfan Xing, Ruiping Zhang, Jinxian Huang

**Affiliations:** ^1^Rheumatology Department, Shenzhen Hospital, The University of Hong Kong, Shenzhen, China; ^2^Microbiology Department, Shenzhen Hospital, The University of Hong Kong, Shenzhen, China; ^3^Pathology Department, Shenzhen Hospital, The University of Hong Kong, Shenzhen, China

**Keywords:** *Salmonella*, next-generation sequencing, acute acalculous cholecystitis, appendicitis, gout

## Abstract

Non-typhoidal *Salmonella* (NTS) disease is not common as typhoid fever but has become a global public health problem in recent decades. Acute acalculous cholecystitis (AAC) and appendicitis are rare complications of NTS infection, which are usually difficult to be diagnosed with atypical signs. Pathogenesis of NTS-induced AAC and NTS-induced appendicitis is still unclear. Ultrasound is the first choice for diagnosis of these two rare complications, computed tomography can assist in and next-generation sequencing (NGS), as a new technology in clinical medicine, also facilitates diagnosis. We described a case of simultaneous AAC and appendicitis due to NTS in an elderly male and further confirmed the diagnosis using NGS. As far as we know, this is the first Asian case of two complications occurring at the same time. Our aim is to alert physicians to pay attention to this rare condition.

## Introduction

*Salmonella* was named after pathologist Salmon over a century ago. The best-known pathogens are *Salmonella typhi* and *Salmonella paratyphi*, the etiologies of enteric fever. Non-typhoidal *Salmonella* (NTS) disease is not most common and generally manifests as self-limiting diarrhea, whereas invasive NTS (iNTS) disease could be fatal and presents as non-specific fever with symptoms that are clinically indistinguishable from other febrile illnesses (1). The definitive diagnosis relies on the isolation of *Salmonella* from normally sterile clinical samples, usually blood and bone marrow. Culture confirms the diagnosis and provides an isolate for antimicrobial susceptibility testing (2). Invasive NTS disease commonly presents as a febrile bacteremia, which requires intravenous antibiotic therapy of 7–14 days' duration. Localized infections often require surgical debridement along with antibiotics, and the course of therapy is dependent on the site of involvement (3).

Acute acalculous cholecystitis (AAC) is defined as acute inflammatory process of the gallbladder without evidence of gallstones contributing to 5–10% of all cholecystitis (4). Acute acalculous cholecystitis is typically seen in hospitalized patients with sepsis, burns, and trauma; those with prolonged use of total parenteral nutrition; and older and immunosuppressed patients (5). Many pathogens including bacteria, yeasts and molds, viruses, and parasites are associated with AAC (6). Mechanisms include bile stasis, total parenteral nutrition, gallbladder ischemia, and vasoactive mediators (6). Appendicitis is the most common condition of an acute abdomen requiring emergency surgery. *Salmonella* may cause infection mimicking appendicitis through mesenteric lymphadenopathy, but is rarely associated with appendicitis (7).

To our knowledge, this is the first adult case of synchronous gangrenous AAC and appendicitis due to NTS infection in Asia. This case highlights the need for more widespread recognition of these rare complications of iNTS diseases and provides strong evidence that NTS attacks the gallbladder and appendix directly through next-generation sequencing (NGS) technology.

## Case

The 90-year-old Chinese gentleman presented to our hospital with progressively worsening left knee joint and left ankle pain for 1 week, accompanied by mild lower abdominal pain and constipation for 2 days. Medical history included 10 years of gout with irregular treatment, hypertension, chronic kidney failure (CKD) stage 3, and permanent pacemaker implantation due to atrial fibrillation. Apart from eating oysters 1 week before admission, he had not eaten anything different from the usual.

On admission, his vital signs were normal. Physical examination revealed swollen left knee and left ankle with tenderness and a soft abdomen with tenderness of right lower quadrant without rebound tenderness. Murphy's sign was negative. Laboratory tests showed that white blood cell (WBC) count, percentage of neutrophils, and C-reactive protein (CRP) were mildly elevated, and mild transaminitis was also identified (Table 1). Stool routine was normal. After intestinal obstruction was excluded by abdominal radiology, he was treated with an intramuscular injection of 7 mg compound betamethasone once for gout attack and 30 mL oral lactulose for constipation.

**Table 1:** Summary of all relevant investigations results.

**Investigations**	**Day 1**	**Day 2**	**Day 4**	**Day 5**	**Normal range**
^a^WBC(10/L)	12.28	15.39	12.56	14.39	(3.89–9.93)
^b^NEUT(%)	78.6%	92.3%	90.9%	90.6%	(44.0–72.0)
^c^CRP(mg/L)	27.85	289.46	332.92	214.47	(0–5)
^d^PCT(ng/ml)	–	144.59	168.61	83.32	–
^e^ALT(U/L)	33.4	–	33.1	28.2	(0–41)
^f^AST(U/L)	24.9	–	35.6	34.4	(0–40)
^g^GGT(U/L)	60.9	–	87.5	104.5	(0–60)
^h^ALP(U/L)	59	–	46	62	(40–130)
^i^TBil(μmol/L)	11	–	5.4	6.4	(0–21)
^j^CREA(μmol/L)	104	367	414	282	(62–106)

a WBC, White blood cell count;

b NEUT, Neutrophil;

c CRP, C-reactive protein;

d PCT, Procalcitonin;

e ALT, Alanine transaminase;

f AST, Aspartate transaminase;

g GGT, Gamma glutamyl transferase;

h ALP, Alkaline phosphatase;

i TBil, Total bilirubin;

j *CREA, Creatinine*.

In the subsequent 2 days, he appeared with persistent diarrhea and ongoing lower abdominal pain with fever of up to 39.4°C. Repeat investigations recognized markedly elevated WBC count, CRP, and procalcitonin, and creatine increased significantly with a peak value of 414 μmol/L (Table 1). The stool routine revealed yellow watery stool with 1 to 3 WBCs per high-power field, and *Clostridium difficile* test was negative. Blood and stool cultures were pending; thus, intravenous ceftriaxone sodium 2 g daily was started empirically. Shortly afterward, blood and stool cultures revealed *Salmonella* group D with resistance to ampicillin and sensitivity to chloramphenicol, trimethoprim/sulfamethoxazole, ceftriaxone, and azithromycin. Meanwhile, although with enough fluid intake, his blood pressure sharply dropped to 88/52 mm Hg. Considering septic shock, treatment was upgraded to intravenous meropenem 1 g every 12 h (half dosage concerning renal insufficiency).

**Figure 1:**
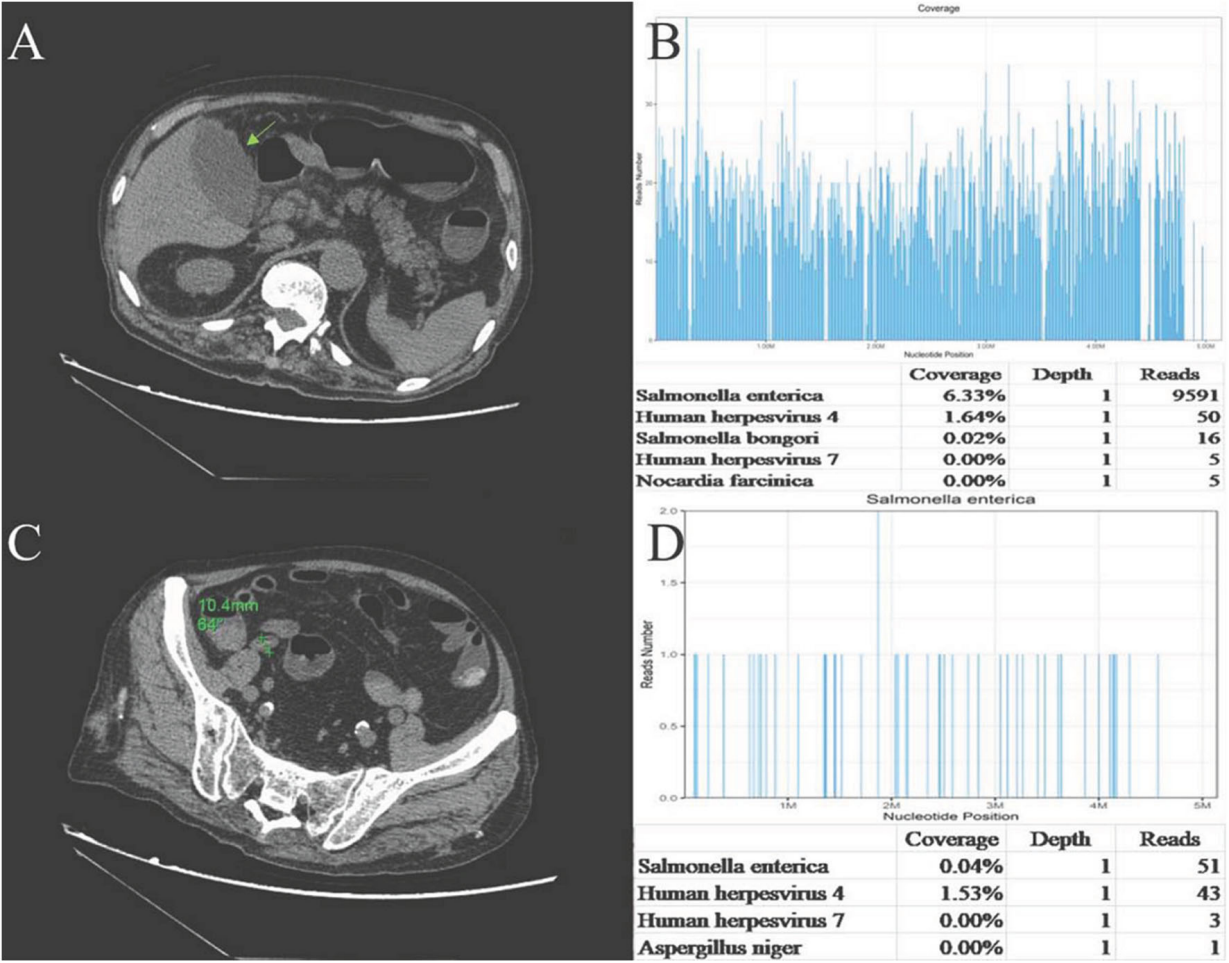
**(A)**Abdominal CT plain: the gallbladder enlarged, and gallbladder wall was thickened and blurred. **(B)** Abdominal CT plain: The appendix cavity thickened, and the widest diameter was ~11 mm. **(C)** Sequencing of *Salmonella* yielded a total coverage of 6.33% in the gallbladder tissue by NGS. Number of unique reads, coverage, and depth of the identified pathogen sequences. The obtained sequence information was compared and analyzed with the known microbial database of NCBI gene bank to determine the type and content of microorganisms in the sample. The more segments detected, the more coverage, and the more reliable the result. **(D)** Sequencing of *Salmonella* yielded a total coverage of 0.04% in the appendix tissue by NGS. Number of unique reads, coverage, and depth of the identified pathogen sequences.

**Figure 2:**
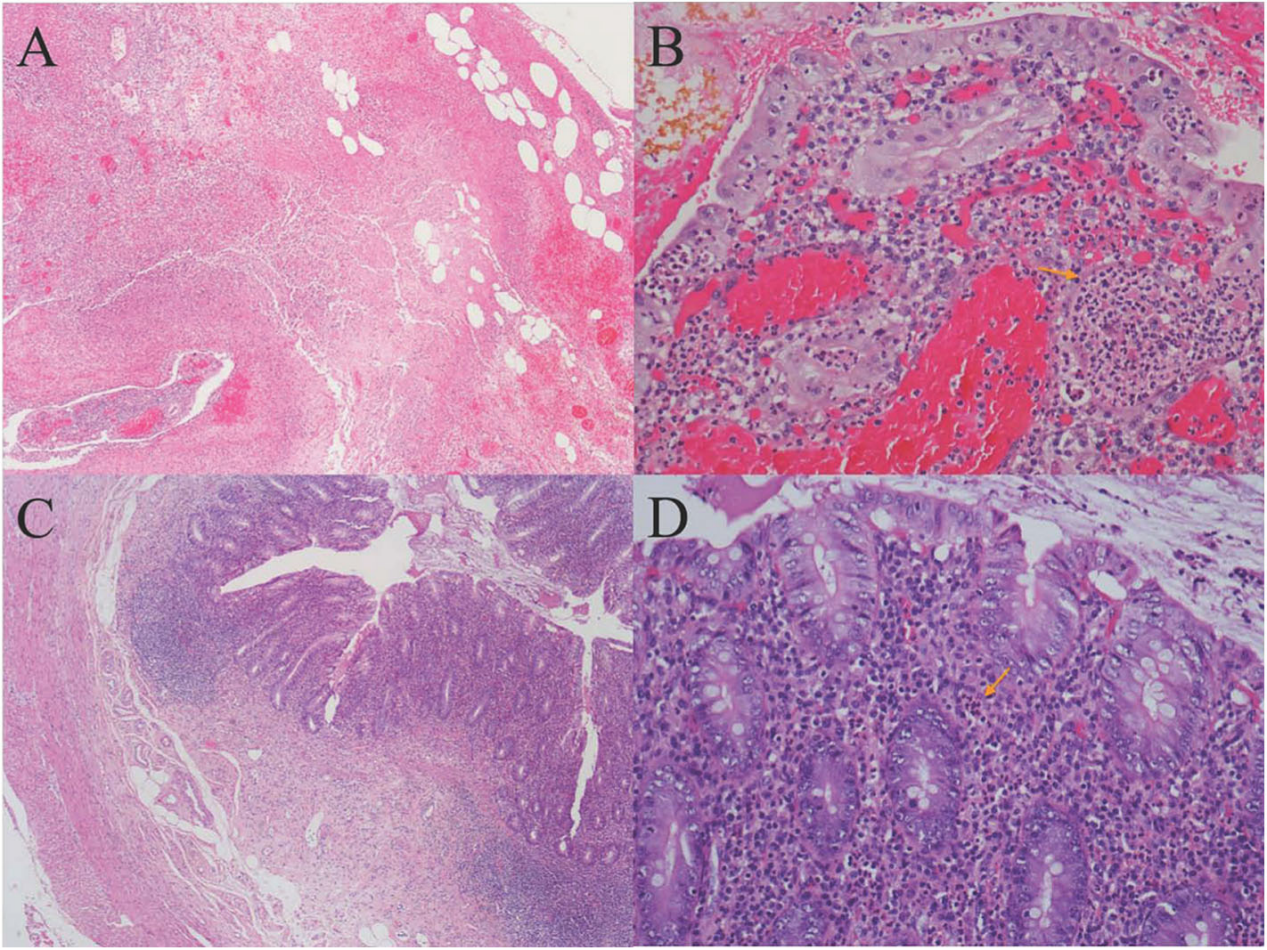
**(A)** Histology of gallbladder tissue (hematoxylin-eosin stain, magnification ×40); **(B)** Histology of gallbladder tissue (hematoxylin-eosin stain, magnification ×200): Extensive transmural infiltration of neutrophils and mononuclear inflammatory cells with intramural abscess formation (yellow arrow) indicating full-thickness necrosis, which contributed to the pathological diagnosis of severe acute gangrenous cholecystitis. **(C)** Histology of appendix tissue (hematoxylin-eosin stain, magnification ×40): The infiltration of both lymphocytes and plasma cells in the appendix wall, accompanied by hyperplasia of fibrous and adipose tissue in the submucosa, revealing chronic inflammatory changes of appendix. **(D)** Histology of appendix tissue (hematoxylin-eosin stain, magnification ×200): The focal inflammatory cell infiltration of neutrophils in the submucosa (yellow arrow) revealed focal acute changes of appendicitis.

One day later, his temperature returned to normal, and diarrhea improved, whereas abdominal pain was not resolved. Physical examination revealed abdominal tenderness without definite location and no rebound tenderness, and Murphy's sign was positive. Bedside ultrasound (US) suggested enlarged gallbladder, sludge formation, and no gallstones were identified. The abdominal computed tomography (CT) scan revealed acute cholecystitis and appendicitis (Figures 1A,C). Then he was transferred to gastrointestinal surgery and underwent laparoscopic cholecystectomy, appendicectomy and enterolysis. Postoperative pathology revealed acute gangrenous cholecystitis (Figures 2A,B) and chronic appendicitis with focal acute inflammation changes (Figures 2C,D). Bile culture was positive for *Salmonella* group D. He recovered after surgery and was discharged after 5 days of intravenous ceftriaxone and metronidazole.

Pathological sections of gallbladder and appendix tissues were sent for NGS analysis (see Supplementary Material for details). The results identified 9,591 *Salmonella enterica* reads in gallbladder tissue, covering 6.33% of the nucleotide sequences, and 51 *S. enterica* reads in appendix tissue, covering 0.04% of the nucleotide sequences. Epstein-Barr virus (EBV, HHV-4) reads of 50 and 43 were also detected in the gallbladder and appendix tissues, respectively (Figures 1B,D). EBV-encoded RNA (EBER) by *in situ* hybridization for appendix tissue was negative.

## Discussion

In recent decades, iNTS disease has become a major public health concern, especially in sub-Saharan Africa. One systematic review extracted case fatality rate (CFR) data for iNTS disease from 24 studies, which estimated an average CFR of 20.6% in Africa (8). The global burden of iNTS disease reflected by all-age CFR was 14.5% in a systematic analysis in 2017, with higher estimates among children younger than 5 years, elderly people older than 70 years, and those with human immunodeficiency virus (HIV) and low sociodemographic index settings. In the same year, the estimated CFR of typhoid and paratyphoid fever was only 0.95% (1). Moreover, one study in Vietnam provided the largest description of iNTS disease cases in Southeast Asia to date, highlighting similarities in iNTS disease epidemiology between Asia and Africa (9). Therefore, iNTS disease might also be a threat in Asia.

For sources and modes of transmission of NTS in industrialized countries, except food contaminated with animal feces, more important sources have been hypothesized, including waterborne transmission, transmission directly from animals and their environments, transmission between people, and independent of a non-human animal reservoir (2). Clinical symptoms associated with acute gastroenteritis caused by NTS infections are often indistinguishable from those caused by other enteric bacterial pathogens, and gold standard for diagnosis still requires isolation of the pathogen from stool samples. The pathogen may also be isolated from the blood, lymph nodes, bone marrow, and other systemic sites (10).

Invasive NTS disease associates with several host risk factors, including gastric acid reduction, extremes of age, and many forms of immunocompromise, such as chronic granulomatous disease. There is also an overwhelming association of iNTS with advanced HIV disease among Africa adults (11–13). The clinical presentation of NTS infection is non-specific among adults. Respiratory symptoms are frequently present, and diarrhea is often not a prominent feature. Features on physical examination include abnormal respiratory findings, such as rapid respiratory rate or chest crepitations suggestive of pneumonia, and hepatosplenomegaly in 30–45% of cases (2). Invasive NTS may lead to localized infections in 5–10% of cases including meningitis, endocarditis, pneumonia, empyema, abscess formation, osteomyelitis, and septic arthritis and may also induce endovascular complications (3). Gastrointestinal manifestation may include hepatomegaly, splenomegaly, cholecystitis, cholangitis, and splenic and hepatic abscesses, but these are uncommon (14).

Invasive NTS disease requires intravenous antibiotic therapy because of increasing reports of resistance; the choice should be based on sensitivity testing of the colonizing isolate. In Southeast Asia, cephalosporin resistance has been reported from Singapore, the Philippines, and Thailand (15, 16). Some of the cephalosporin-resistant iNTS cases in Thailand also show resistance to fluoroquinolones (17, 18). Furthermore, local infections “that do not improve after antibiotic treatment may require endoscopic or surgical therapy.

Our case with quite a few risk factors, including extreme age, CKD stage 3, and long-term abnormal uric acid metabolism, can be defined as immunocompromised, who was susceptible to *Salmonella* infection. In recent years, high rates of *Salmonella* contamination of retail meats have been reported in China (19). The *Salmonella* in our case most likely originated from contaminated seafood or meat products. The patient had constipation only on admission, but fever, abdominal pain, and diarrhea in the following days, which were all common symptoms of NTS infection. The diagnosis of NTS bacteremia was confirmed by the isolation of *Salmonella* group D in blood and stool cultures. Improved pyrexia, decreased inflammatory markers, and subsequent susceptibility tests have all demonstrated that the empirical ceftriaxone is sensitive and effective, even the patient lived in a region with high rates of cephalosporin-resistant NTS.

Acute acalculous cholecystitis is the most frequent form of acute cholecystitis in children, compared with only 5–10% for all adult cases (20). Lothrop (21) reported the first case of AAC as a complication of salmonellosis. However, the mechanisms of *Salmonella*-induced AAC are still not clear, and most plausible hypotheses to date are (a) the endotoxin-mediated reaction, which leads to bile stasis increase of bile viscosity, sludge formation, and finally gallbladder mucosal damage; (b) bacteria through portal vein directly attack the biliary system; (c) the lymphatic drainage from gastrointestinal tract; and (d) retrograde biliary carriage (22). The diagnosis of AAC as a complication of NTS infection is based on clinical, laboratory, radiological findings and also requires the isolation of NTS in stool or blood cultures (23). Abdominal US is the test that should be performed first for every case of suspected acute cholecystitis. Ultrasound is recommended as the most accurate modality to diagnose acalculous cholecystitis (24). The criteria of AAC include thickening of the gallbladder wall (3 mm or greater), pericholecystic fluid, direct tenderness when the probe is pushed against the gallbladder (ultrasonographic Murphy's sign), enlarged tense gallbladder, and absence of gallstones. Computed tomography and hepatoiminodiacetic acid scan are considered a good adjunct to US (25). Generally, if there are no any complications, such as empyema, gangrene, or perforation of the gallbladder, conservative treatment is sufficient. Otherwise, the surgical treatment is mandatory (25). The AAC in our case was grade II (moderate) acute cholecystitis according to Tokyo Guideline (26); thus, laparoscopy was mandatory.

There were 9,591 *S. enterica* reads and 50 EBV reads detected in gallbladder tissue through NGS technology. *Salmonella* is known to colonize in bile ducts and the gallbladder in some cases, even after apparent recovery (27). Therefore, we speculated that the mechanism of AAC in this case may be hematogenous transmission, rather than gallbladder ischemia or cholestasis secondary to sepsis, that is, bacteria retrograded from portal vein to the gallbladder vein, and then colonize in the gallbladder and directly attack the gallbladder, which caused focal inflammation and mucosal damage eventually. Blood, stool, and bile cultures helped to confirm the diagnosis of NTS-induced AAC, and the large number of *Salmonella* sequences detected by NGS provided strong evidence and demonstrated that NTS attacked the gallbladder directly.

A small number of EBV sequences were also detected in the gallbladder. Although as one of the frequent viruses associated with AAC, EBV is usually not considered to be the cause of cholecystitis (28). Epstein-Barr virus infection can lead to acute infectious mononucleosis characterized by fever, sore throat, and lymphadenopathy. Elevated liver function and hepatosplenomegaly may also be present. The patient did not have any of these clinical manifestations or abnormal liver function. Epstein-Barr virus is estimated to infect more than 98% of adults worldwide and is one of the most common human viruses (29). The patient might have latent infection of EBV, which was not considered to be the cause of AAC. There were also 16 reads of *Salmonella bongori* detected in gallbladder tissue, probably because of homogenous overlap with *S. enterica*. Other detected sequences were of little significance and were considered as contamination.

Appendicitis is a common acute abdominal disease that generally requires prompt surgical intervention to minimize morbidity and mortality. Most often there are cases of *Salmonella* infections with signs and symptoms mimicking those of appendicitis (7). Appendicitis is a rare form of presentation of acute abdomen in *Salmonella* infections (30). The mechanism of *Salmonella*-induced appendicitis could be pathogen predilection for the appendix or invasion of the appendix from the portal venous system (31). Ultrasound should be used first in patients suspected of having appendicitis, and CT scans should be used judiciously (32). *Salmonella* sequences detected by NGS in our case also demonstrated that NTS might attack the appendix directly. Similar number of EBV sequences was also detected in the appendix. However, except for fever and leukocytosis, the patient did not have any typical manifestation of EBV infection, such as elevated liver function tests, lymphadenectasis, or hepatosplenomegaly. The pathology of appendix revealed focal inflammatory cell infiltration of neutrophils, which generally appears in bacterial infection, rather than in viral infection. The histologic findings of EBV infective enteritis show transmural inflammation with extended lymphoid infiltration, fissuring ulcers, and intraepithelial lymphocytosis (33), which were not found in this case. Recovery of our case after antibacterial therapy and the absence of EBV existence in the appendix as detected by EBER, indicated that EBV was not the cause of appendicitis. EBV antibodies, DNA testing, and EBV-encoded RNA (EBER) were not performed then because EBV existence was merely detected by NGS, which might be a limitation for this case to further exclude EBV infection.

The sequence reads in the appendix were much lower than those in the gallbladder. We speculate several possible explanations: First, *Salmonella* may have a positional preference for organs, attacking the gallbladder first and later the appendix, which leads more bacteria to colonize the gallbladder than the appendix. Second, the local drug concentration of meropenem in the appendix may be higher than that in the gallbladder, killing more bacteria and resulting in lower quantities of remaining pathogens. Finally, there was a 1-week interval between sequencing, that is, the gallbladder first and then the appendix. Storage with DNA degradation may also affect the sequencing results. In addition, administration with sensitive antibiotics before sampling contributed to the relatively lower determination of reads in both tissues.

We searched the PubMed database using the search words “*Salmonella*” and “appendicitis” and compared our case with previously published case reports. The search resulted (Table 2) in seven reported cases of NTS-induced appendicitis. Dadswell (34) was the first to report NTS associated with appendicitis. The report mentioned two cases in which *Salmonella enteritidis* was isolated from stool, but no other information was provided. The remaining cases included three pediatric patients, two elderly patients, and one adult patient with a history of kidney transplantation, all of whom belonged to immunocompromised population. All six patients had typical clinical symptoms, and the diagnosis of appendicitis could be confirmed by imaging or intraoperative findings. White blood cell count and creatinine level were not necessarily elevated (three within normal range in six reported cases). All had good prognosis after surgical treatment and postoperative antibiotic therapy or conservative treatment with antibiotics alone. Duration of postoperative antibiotic treatment could be diversified without guidelines to follow so far. Non-typhoidal *Salmonella* was isolated from various specimens in all cases, including blood, stool, and pus, but the association between appendicitis and NTS infection still remained unclear. In our case, blood and stool cultures confirmed the diagnosis of iNTS disease, whereas the detection of *S. enterica* in the appendix confirmed the definitive diagnosis of NTS-induced appendicitis.

**Table 2:** Characteristics of the previous and present cases of NTS -related appendicitis.

**Authors**	**Country**	**Number of cases**	**Age**	**Gender**	**PMH**	**Clinical symptoms**	**Physical examination**	**WBC**	**Creatine**	**Positive culture**	**Pathogens**	**Appendicectomy**	**Antibiotics**
Dadswell (34)	Britain	2	–	–	–	–	–	–	–	Stool	Salmonella enteritidis	–	–
Bartoli et al. (35)	Switzerland	1	12	Female	In good health	Fever, mild abdominal pain, vomiting, watery diarrhea	Rebound tenderness	–	–	Intraoperative	Salmonella enterica serovar Israel	Yes (laparotomy)	Ceftriaxone (post-operation)
Malone et al. (36)	USA	1	33	Male	Kidney transplant 7 years ago, maintenance oral immunosuppression	Fever, epigastric pain, vomiting	Right lower quadrant (RLQ) tenderness, guarding and rebound	8.5 × 10^9^/L	Slightly increased (177μmol /dL from baseline)	Blood	Salmonella group B	Yes (laparoscope)	5 days of unknown antibiotics (post-operation)
Stewart-Parker et al. (30)	Britain	1	10	Male	Healthy	Fever, right lower quadrant pain, nausea	Soft abdomen with mild tenderness in the right iliac fossa (RIF)	16.7 × 10^9^/L	–	Pus	Salmonella enteritidis	Yes (laparoscope)	7 days of intravenous Ceftriaxone (post-operation)
Wong et al. (37)	Singapore	1	78	Male	Ischemic heart disease (IHD), chronic obstructive pulmonary disease (COPD), bronchiectasis, peptic ulcer disease, hypertension hyperlipidaemia	Fever, cough, diarrhea, vomiting	Bilateral crepitations in the lung	12.8 × 10^9^/L	Acute kidney injury (118 μmol/L)	Blood stool	NTS species	No	2 weeks of intravenous Ceftriaxone (after culture result reported)
Joseph et al. (38)	Australia	1	13	Male	Healthy, recently travelled to Bali, Indonesia	Fever, worsening right-sided abdominal pain, diarrhea, vomiting	Tachycardic and peritonism of the right lower quadrant	8.0 × 10^9^/L	–	Stool	Salmonella serotype B	Yes (laparoscope)	7 days of oral Azithromycin (post-operation)
Present case	China	1	90	Male	Gout, hypertension, permanent pacemaker implantation due to atrial fibrillation, chronic kidney failure (CKD) stage 3	Fever, constipation, watery diarrhea, abdominal pain	RLQ tenderness, no rebound	12.3 × 10^9^/L	Acute kidney injury (118 μmol/L)	Blood, stool, bile gallbladder appendix tissues	Salmonella group D	Yes (laparoscope)	2 days of Meropenem (pre-operation) 5 days of intravenous Ceftriaxone and Metronidazole (post-operation)

## Conclusion

Invasive NTS disease is a major public health problem on a global scale, with high morbidity and mortality. The burden of iNTS disease in Asia has been underestimated because of a lack of attention and surveillance. Acute acalculous cholecystitis and appendicitis are rare complications of NTS infection, and the diagnosis is challenging. We described a case of simultaneous AAC and appendicitis due to NTS in an adult patient and further confirmed the diagnosis using NGS. This case gave hints and warning to physicians. If iNTS disease is suspected, blood and stool cultures should be delivered. Once a patient is suspected of having AAC and appendicitis, US is the most rapid and reliable diagnostic method to use. Antibiotic treatment should be started immediately and adjusted according to drug sensitivity test. If symptoms do not improve, surgery should be performed immediately. More researches on mechanisms of appendicitis and cholecystitis due to NTS are needed.

## Data Availability Statement

The raw data supporting the conclusions of this article will be made available by the authors, without undue reservation.

## Ethics Statement

Written informed consent was obtained from the individual(s) for the publication of any potentially identifiable images or data included in this article. Written informed consent was obtained from the patient for publication of this case report.

## Author Contributions

YZ and JH set the conceptual design of the study, data collection, and made the first draft of the manuscript. LZ, FX, and JH performed clinical care, diagnosis, and analysis on patients' status. RZ performed the pathological procedure and provided the images. All authors contributed to the article and approved the submitted version.

## Conflict of Interest

The authors declare that the research was conducted in the absence of any commercial or financial relationships that could be construed as a potential conflict of interest.
