# Supplementary material for: Synchronous Acute Acalculous Cholecystitis and Appendicitis Due to Salmonella Group D: A Rare Case Report From China and Review of the Literature
Source: Front Med (Lausanne). 2020 Aug 18;7:406. doi: 10.3389/fmed.2020.00406 (PMC7461861; doi:10.3389/fmed.2020.00406)
Supplement: Supplementary file 1 [file Table_1.DOCX]

**Supplementary material**

**Next generation sequencing (NGS) procedures**

**Sample processing and DNA extraction:** Selected areas from the paraffin mass of tissues and 10 slices were prepared for testing. Totally 4 slices were selected and appropriate amount of xylene was added for process of dewaxing. Nucleic acid was extracted from the dewaxed samples by Kajie automatic extractors using QIAsymphony DSP Virus/Pathogen Midi Kit. Concentration was measured using Qubit3.0 Fluorometer and the sample was diluted with nuclease free water to 1ng/μL.

**Construction of DNA libraries:** The obtained nucleic acids were further processed with TruePrepTM DNA Library Prep Kit V2 for lllumina and then were added with marker sequences. The above products were introduced into the sequencing joint through PCR amplification, and Qubit quantification of the sequencing library was performed after purified by Agencourt AMPure XP beads.

**Sequencing:** The library was sequenced on NextSeq 500 as required. The sequencing strategy was SE75, and the data volume was over 10M reads.

**Bioinformatic analysis:** The obtained sequence information was compared and analyzed with the known microbial database of NCBI genebank to determine the type and content of microorganisms in the sample.
